# The prognostic value of immune-related genes *AZGP1*, *SLCO5A1*, and *CTF1* in Uveal melanoma

**DOI:** 10.3389/fonc.2022.918230

**Published:** 2022-08-16

**Authors:** Wanpeng Wang, Sha Wang

**Affiliations:** ^1^ Eye Center of Xiangya Hospital, Central South University, Changsha, China; ^2^ Hunan Key Laboratory of Ophthalmology, Changsha, China; ^3^ National Clinical Research Center for Geriatric Disorders, Xiangya Hospital, Changsha, China

**Keywords:** bioinformatics, uveal melanoma, immunity, prognosis, UM

## Abstract

**Objective:**

Uveal melanoma (UM) is an aggressive malignancy with a poor prognosis and no available effective treatment. Therefore, exploring a potential prognostic marker for UM could provide new possibilities for early detection, recurrence, and treatment.

**Methods:**

In this study, we used “ConsensusClusterPlus” to classify patients with UM into subgroups, screened for significant differences in immune prognostic factors between subgroups, selected three genes using LASSO (Least absolute shrinkage and selection operator) regression to construct a risk model, and performed tumor immune cell infiltration analysis on the risk model. infiltration analysis, and then verified the heterogeneous role of the 3 core genes in other cancers by pan-cancer analysis and validate its expression by RT-qPCR in normal and tumor cells.

**Results:**

We consistently categorized 80 UM patients into two subgroups after the immunogenetic set, where the UM1 subgroup had a better prognosis than the UM2 subgroup, and used 3 immune-related genes AZGP1, SLCO5A1, and CTF1 to derive risk scores as independent prognostic markers and predictors of UM clinicopathological features. We found significant differences in overall survival (OS) between low- and high-risk groups, and prognostic models were negatively correlated with B cell and myeloid dendritic cell and positively correlated with CD8+ T cell AZGP1 and CTF1 were significantly upregulated in UM cells compared with normal UM cells.

**Conclusion:**

Immunogens are significantly associated with the prognosis of UM, and further classification based on genetic characteristics may help to develop immunotherapeutic strategies and provide new approaches to develop customized treatment strategies for patients.

## Introduction

Uveal melanoma (UM) is a malignancy of pigment cell derivation occurring in the eye with a poor prognosis and susceptibility to metastasis, as well as a mortality rate of up to 50% (1, 2). Tumor metastasis can occur at any time (3). Once metastases are detected, the median survival time of UM patients is about 12 months (4, 5). Therefore, the search for a stable clinical indicator and molecular biomarker that can predict patient prognosis has become a hot topic in the UM treatment domain (6).

The tumor microenvironment (TME) has been found to play a key role in cancer progression and treatment response (7, 8). Prognostic or predictive biomarkers associated with the TME may hold great promise in identifying molecular targets and guiding patient management. The current therapeutic options for the treatment of metastatic UM include liver directed therapies and systemic targeted immunotherapy. Notably, immunotherapy may be a potential option for alternative or adjuvant therapy, even in prophylactic settings. High-risk UMs that metastasize usually contain macrophages and lymphocytes. These lymphocytes are usually regulatory T cells that can suppress the immune response, but UM may be particularly sensitive to T cell-based immunotherapy. Another treatment option for patients with metastatic UM is targeted therapy with T cells that target tumor-associated antigens. Some studies have demonstrated the potential role of PRAME (preferentially expressed antigen in melanoma)-targeted immunotherapy in patients with metastatic UM (9). Although substantial progress has been attained in some important genes and pathways involved in the diagnosis and treatment of UM, the prognosis of UM is still poor. Therefore, there remains an urgent need to develop an immune-related prognostic marker for UM.

Unsupervised class discovery is a data mining technique used to detect unknown groups of possible items based on intrinsic features and no external information. Consensus clustering methods provide quantitative and visual stability evidence for estimating the number of unsupervised classes in a dataset (10). In recent years, consensus clustering has been increasingly used in cancer research to classify cancers into different subgroups by consensus clustering and to explore the differences in clinical features and heterogeneous expression of genes between subgroups. Twenty-two common epithelial–mesenchymal transition (EMT)-related genes that were differentially expressed in gliomas were divided into two subgroups by consensus clustering, and then seven EMT-related genes were used to derive risk scores as independent prognostic markers and predictors of glioma clinicopathological features (11). In lung adenocarcinoma (LUAD), molecular subtypes of LUAD were identified based on tumor invasion-related genes and a 5-gene signature prognostic stratification system (12). Therefore, different UM subgroups can be developed by consensus clustering to further explore immunotherapeutic strategies.

This study aimed to classify patients with UM into subgroups based on immune gene sets using a hierarchical clustering approach to screen prognostic factors by taking the intersections of differentially expressed genes (DEGs) and immune-related genes between subgroups for prognostic analysis and then constructing prognostic risk models by LASSO to obtain the three core immune prognostic genes AZGP1, SLCO5A1, and CTF1. These were subsequently subjected to pan-cancer analysis to verify their heterogeneous roles in other cancers of this kind and to come up with new targets for the diagnosis and treatment of UM.

## Materials and Methods

### Data sources and statistical methods

Tumor RNAseq (RNA-sequencing) data were obtained from The Cancer Genome Atlas (TCGA) database (IlluminaHiSeq: log2-normalized_count+ 1). A list of immune-related genes was rooted in the Immunology Database and Analysis Portal (ImmPort) database. Data on immune infiltrating cell scores in six from UM were downloaded from the TIMER database. Subsequent statistical methods were implemented using the R software without special instructions.

### Hierarchical clustering

UM patients were divided into different subgroups according to the expression of the immune gene set and the relationship between different subgroups, and immunity was observed. The maximum number of clusters was 6. The optimal number of clusters was inferred by choosing the appropriate K value, and the clustering heat map was analyzed using the R package pheatmap. The immune infiltration was reliably estimated using the R packages ggplot2 and pheatmap, and the distribution of immune checkpoint-associated genes was observed.

### Functional enrichment analysis

Differential analysis of immune-related genes was constructed using the R package limma, with *p* < 0.05 and |fold change|>2 as the screening conditions represented by a volcano plot. Meanwhile, the intersection of the screened DEGs with immune-related genes was taken using Venn diagrams. Gene Ontology (GO) functional enrichment and Kyoto Encyclopedia of Genes and Genomes (KEGG) pathway analysis of the intersected genes were performed using the R package cluster profile. For gene set functional enrichment analysis, the h.all.v7.4.symbols.gmt subset was downloaded from the Molecular Signatures Database (http://www.gsea-msigdb.org/gsea/downloads.jsp) and used as a background to map genes to the The enrichment analysis was performed using the R package clusterProfiler (version 3.14.3) to obtain the results of gene set enrichment. A minimum gene set of 5 and a maximum gene set of 5,000 were set, and a *p* value <0.05 and an FDR <0.25 were considered statistically significant.

### Survival analysis

The RNAseq data (level 3) and corresponding clinical information were collected from patients with UM in TCGA database. Log-rank was used to test the difference in survival between the two parts mentioned above for Kaplan–Meier (KM) survival analysis. For the KM curves, *p*-values and hazard ratios (HRs) with 95% confidence intervals (CIs) were derived *via* log-rank test and univariate Cox regression. *p* < 0.05 was considered significant.

### Prognostic signature model

The relationship between prognostic immune-related gene expression and overall survival (OS) was first assessed. A prognostic risk prediction model for UM was developed. UM patients were divided into high- and low-risk groups using the median risk score as a cutoff. KM curves were plotted to compare the OS between the high- and low-risk groups. The reactive oxygen species (ROC) survival analysis was performed, and the decision curve analysis was performed using the “rmda” package. The relationship between the risk score model and tumor immune infiltrating cells was also investigated using and found to be statistically significant at *p* < 0.05.

### Pan-cancer analysis

The RNAseq data (level 3) of different tumor tissues and tumor paracancer tissues were obtained from TCGA database. Prognostic analysis was performed using univariate Cox regression analysis, and the forest plot was used to show the *p*-value, HR, and 95% CI through the “forestplot” R package. The rank-sum test was used to detect the difference between the two data parts, and *p* < 0.05 was significant.

### Cell culture

Human UM cell line OM431 and normal UM cells changed to: Human retinal pigment epithelial cell line (hTERT RPE-1 U1L) were purchased from ATCC (American Type Culture Collection) and cultured in DMEM (Dulbecco's Modified Eagle Medium) containing 100 ml/L fetal bovine serum, 100 U/ml penicillin, and 100 μg/ml streptomycin at 37°C with 5% CO_2_ by volume.

### RT-qPCR

Total RNA was extracted from groups of cells using TRIzol reagent (Invitrogen, Carlsbad, CA, USA) following the manufacturer’s guidelines. cDNA was synthesized from RNA using the PrimeScript RT kit (Takara, Dalian, China). cDNA was amplified and quantified using SYBR Green mix (Takara) in an Applied Biosystems 7500 instrument. The primer sequences used in this study are shown in **Table 1**. -2^ΔΔCt^ method was used to determine the relative gene expression.

**Table 1:** Primer sequences.

Name of primer	Sequences
AZGP1-F	5’-TACAACGACAGTAACGGGTCT-3’
AZGP1-R	5’-TATTTCCAGAATGCTCCGCTG-3’
SLCO5A1-F	5’-TGCCTCTACGTGGTCCTCAC-3’
SLCO5A1-R	5’-TTACGCTGCTCAGGTACCCA-3’
CTF1-F	5’-CCCTCCTCGTCTGCATGGTA-3’
CTF1-R	5’-GAGGCCAAAGGGAACTGAGG-3’
GAPDH-F	5’-CAAGCAACTGTCCCTGAG-3’
GAPDH-R	5’-TAGACAGAAGGTGGCACA-3’

## Results

### Consensus clustering of immune-related uveal melanoma

**Figure 1:**
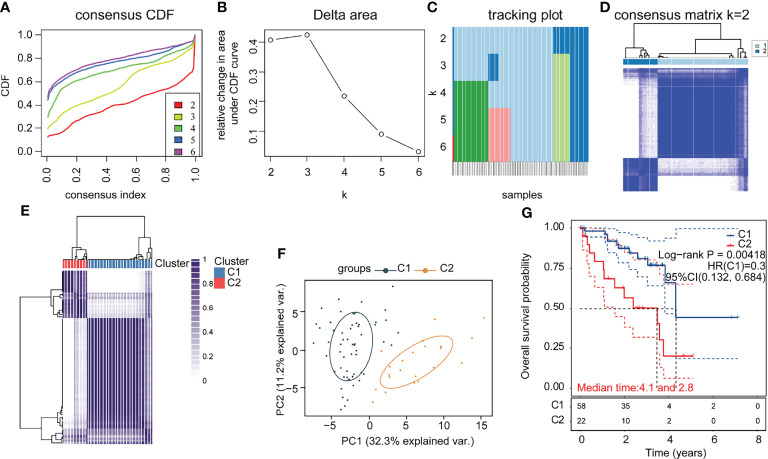
Consensus clustering of immune-related uveal melanoma (UM) **(A)** CDF plot; **(B)** CDF Delta area plot; **(C)** clustered sample distribution plot (the color indicates the sample distribution cohort, the vertical coordinate indicates the number of clusters, and the horizontal coordinate indicates the samples); **(D)** consensus matrix heat map with two sample clusters defined (consensus range of 0–1, with 0 representing white, meaning that the samples are not clustered, and 1 representing blue, meaning that the samples are always clustered); **(E)** sample correlation heat map (range of 0–1 and the larger the value, the higher the correlation); **(F)** PCA (Principal Component Analysis), clustering plot between samples; **(G)** KM (Kaplan-Meier) survival curve of the two subgroups (statistically significant at *p* < 0.05).

To investigate the role of immune-related genes in UM, we used the ConsensusClusterPlus software package to cluster patients according to their immune-related gene expression profiles. The consensus cumulative distribution function (CDF) plots showed a good proportion of disambiguation clusters (**Figures 1A–C**) when the number of clusters was two, dividing the patients into two independent groups (**Figures 1D, E**). We evaluated these two clusters, and the results showed that they remain separable (**Figure 1F**). We compared the differences in prognostic and clinical characteristics of patients belonging to these two UM subtypes. We found that the patients in part 2 had a significantly worse prognosis compared with that of the patients in part 1. A comparison of the clinical baseline information between the two groups revealed significant differences in survival status between the two parts (**Table 2**). Thus, based on the immune gene set, the sample could be divided into two subtypes through consensus clustering. Notably, significant differences in clinical prognosis survival were noted between the two subtypes (**Figure 1G**, *p* = 0.00418).

**Table 2:** Comparison of clinical data between subgroups.

Character	C1	C2	*P*-value
Status			0.004
Alive	47	10	
Dead	11	12	
Mean (SD)	50.8(14.2)	63.8(13.4)	
Age			0.397
Median [MIN, MAX]	63.5[22, 86]	60[37, 86]	
Gender			0.344
FEMALE	23	12	
MALE	35	10	
pT-stage			0.97
T2a	3		
T3	6	2	
T3a	18	6	
T3b	3	1	
T4	1	1	
T4a	17	6	
T4b	7	2	
T4c	1	1	
T4d	1	1	
T4e	1		
T2		1	
T2b		1	
pN-stage			0.646
N0	56	20	
NX	2	2	
pM-stage			0.637
M0	54	19	
M1b	2		
MX	2	2	
M1		1	
pTNM-stage			0.85
IIA	9	3	
IIB	20	7	
IIIA	19	7	
IIIB	8	2	
IV	2	2	
IIIC		1	
New tumor event type			0.903
Metastasis	9	7	
Primary	2	3	
Recurrence	1		
History of neoadjuvant treatment therapy type			
No neoadjuvant	58	22	
Chemotherapy	2	2	

### Analysis of subgroup gene expression differences in immune-related uveal melanoma

**Figure 2:**
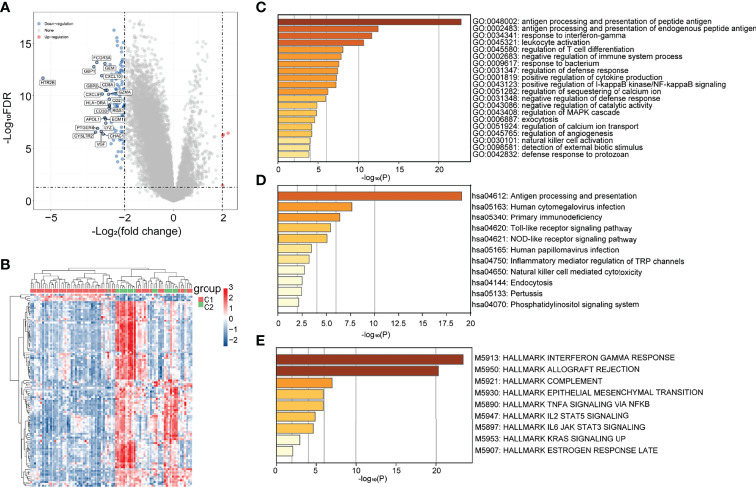
Differential analysis of subgroup gene expression in immune-related uveal melanoma (UM). **(A)** Volcano map; **(B)** heat map; **(C–E)** GO (BP only), KEGG, and Hallmark pathway enrichment maps.

To explore the hidden mechanisms driving the differences in clinical immune characteristics and biological functions between the two subgroups, we analyzed the differences in their mRNA expression profiles in TCGA database. The DEGs were analyzed for immune-related C1 and C2 UM subtypes according to |Log2|FC||>2, adj. *p* < 0.05 as the screening threshold. Four upregulated and 115 downregulated genes were obtained (**Figure 2A**). The heat map shows the upregulated and downregulated DEGs (**Figure 2B**). GO (using BP only), KEGG, and Hallmark were selected as the databases for the functional gene enrichment analysis of these DEGs by Metascape (**Figures 2C–E**). The parameters for enrichment analysis are as follows: the number of Min Overlap genes was 3, the *p*-value cutoff was 0.01, and the Min Enrichment was 1.5.

### Immune-related prognostic model for uveal melanoma

**Figure 3:**
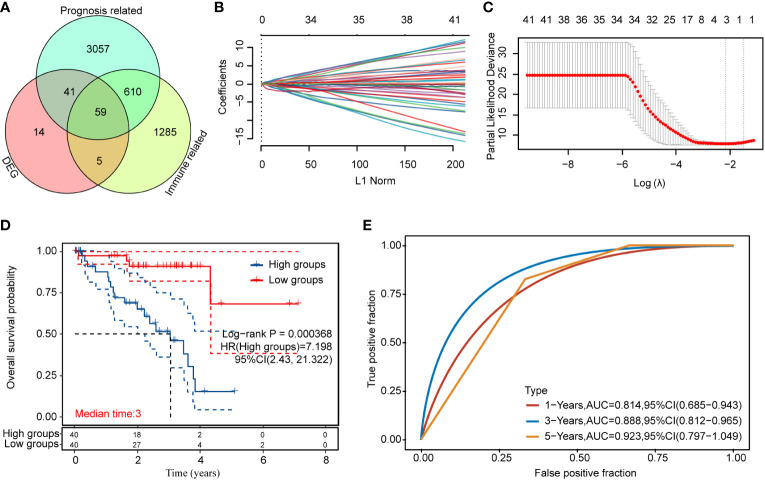
Immune-related prognosis model construction for DEGs **(A)** Intersection of the DEGs, prognosis-related genes, and immune-related genes; **(B, C)** distribution of LASSO coefficients of immune prognosis common genes of DEGs to obtain the adjustment parameter λ.min = 0.000368; **(D)** KM survival curves for high- and low-risk groups; **(E)** ROC curves at 1, 3, and 5 years (statistically significant at p < 0.05).

**Figure 4:**
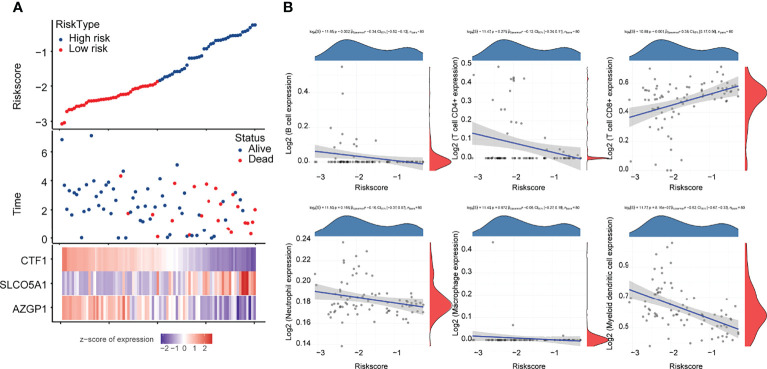
Correlation analysis of the prognostic signature model with tumor immune cell infiltration **(A)** Survival status and duration of patients with UM; **(B)** letters A–G represent B cells, CD4+ T cells, CD8+ T cells, neutrophils, macrophages, myeloid dendritic cells, and uncharacterized cells, respectively. The horizontal coordinates in the figure represent the model score distribution, whereas the vertical coordinates are the immune score distribution. The density curves on the right side represent the immune score distribution trends. The upper side density curve is the trend of distribution of one gene or model score. The uppermost value represents the correlation *p*-value, correlation coefficient, and correlation calculation method, with *p* < 0.05 considered as statistically significant.

We obtained 58 identical genes (**Figure 3A**) by taking the intersection of the subgroup DEGs, prognosis-related genes, and immune-related genes. Then, prognostic characteristics were established (**Figures 3B, C**) for the 58 genes based on LASSO Cox analysis, which were determined by Riskscore = (-0.0548)*AZGP1+(0.0207)*SLCO5A1+(- 0.4091)*. CTF1 divided the patients with UM into high-risk and low-risk parts (**Figure 4A**). Their survival status is shown in **Figure 4B**. There was a significant difference in survival between the high- and low-risk groups (*p* = 0.00368, **Figure 3D**). Prognostic survival prediction was performed for 1, 3, and 5 years, and this prognostic model showed good sensitivity and specificity (**Figure 3E**). Based on the foregoing, AZGP1 and CTF1 were good prognostic genes, and SLCO5A1 was the main risk gene.

Subsequently, we analyzed the correlation between this prognostic signature model and tumor immune cell infiltration (ICI). The results are shown in **Figure 4B**, indicating a negative correlation with B cells and myeloid dendritic cells (*p* < 0.05) and a positive correlation with CD8+ T cells (*p* < 0.05).

### Three core gene prognosis, clinical characteristics, and model accuracy analysis

Based on the prognostic signature model, CTF1, SLCO5A1, and AZGP1 are the core genes of 59 intersecting genes. To verify the clinical significance of these three core genes for UM, we used OS as an index to determine the correlation between gene expression and prognosis. The prognostic relevance was clarified by KM survival curves.

**Figure 5:**
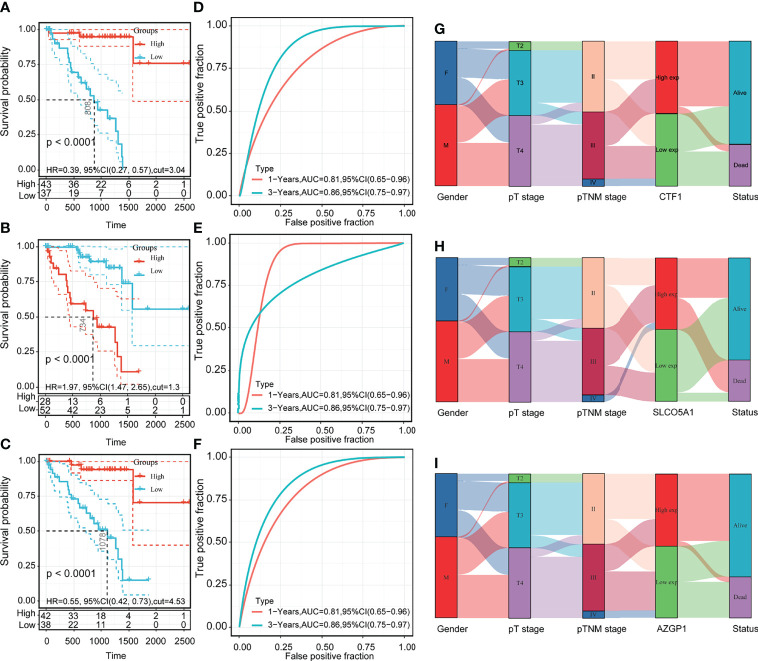
Prognosis of core genes, clinical characteristics, and model accuracy analysis. **(A–C)** KM survival curves of high and low expressions of AZGP1, SLCO5A1, and CTF1; **(D–F)** 1-year and 3-year ROC curves of high and low expressions of AZGP1, SLCO5A1, and CTF1; **(G–I)** Sankey plots of high and low expressions of AZGP1, SLCO5A1, and CTF1 with clinical characteristics; statistically significant at *p* < 0.05.

We used OS as an index to judge the prognostic relevance of gene expression, the KM survival curve to clarify its prognostic relevance, and ROC (subject operating curve) as a tool to judge the accuracy of the model. The results showed that high expressions of AZGP1 and CTF1 had better OS than low expressions in UM (**Figures 5A, C**), and a high expression of SLCO5A1 had worse OS than a low expression (**Figure 5B**). The ROC curve confirmed the accuracy of the model (**Figures 5D–F**). We then further validated the correlation between the high and low expressions of these three genes on clinical characteristics, and the results showed that the high and low expressions of AZGP1, SLCO5A1, and CTF1 were strongly correlated with survival status (**Figures 5G–I**). These results suggest that the expressions of the three core genes AZGP1, SLCO5A1, and CTF1 affect the clinical prognosis of UM.

### Analysis of the correlation between the three core genes and the pathway

To further explore the mechanistic roles of the three core genes in UM, we performed a pathway correlation analysis on the three core genes CTF1, SLCO5A1, and AZGP1. The results showed that the top 20 correlated pathways were mainly focused on immune-related pathway signals, among which CTF1 and AZGP1 were negatively correlated (**Supplementary Tables 1**, **3**) whereas SLCO5A1 was positively correlated (**Supplementary Table 2**).

### Expression of three core genes in uveal melanoma cells

**Figure 6:**
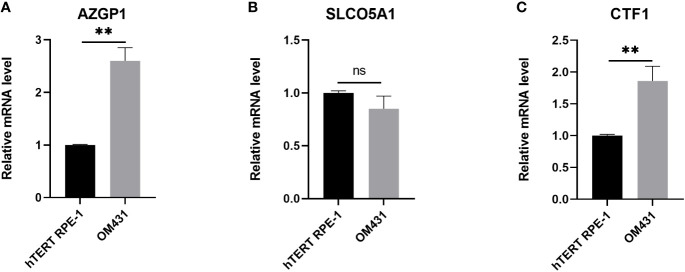
Verification of the expression of the three core genes in cells **(A–C)** The expression of AZGP1, SLCO5A1, and CTF1 in Human retinal pigment epithelial cell line(hTERT RPE-1) and uveal melanoma cells (OM431) was detected by RT-qPCR, cellular experiments were performed in three independent replicates, data in the figures are all measures in the form of mean ± standard deviation, data were analyzed by t-test,** indicates *p* < 0.01.

We then examined the mRNA levels of AZGP1, SLCO5A1, and CTF1 in UM, and the results are shown in **Figure 6**. AZGP1 and CTF1 were significantly upregulated in UM cells compared with hTERT RPE-1 cell (**Figure 6A, C**).

### Pan-cancer analysis of the three core genes

**Figure 7:**
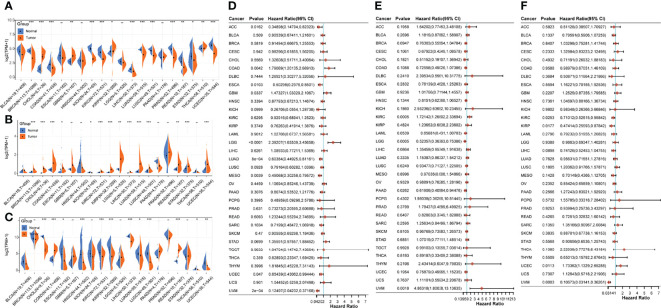
Pan-cancer analysis of core genes. **(A–C)** Expressions of AZGP1, SLCO5A1, and CTF1 in different cancers and paracancerous tissues; **(D–F)**: prognostic survival effects of AZGP1, SLCO5A1, and CTF1 in different cancers; * indicates *p* < 0.05, ** indicates *p* < 0.01, and *** indicates *p* < 0.001, with *p* < 0.05 being significant.

To evaluate the heterogeneous roles of the three core genes in different cancers, we performed a pan-cancer analysis. First, we compared the gene expression profile data of the three genes in different cancer tissues and normal tissues adjacent to the cancer. Compared with the paracancerous tissues, CTF1 was significantly decreased in BLCA(Bladder Urothelial Carcinoma), BRCA(Breast invasive carcinoma), ESCA(Esophageal carcinoma), KICH(Kidney Chromophobe), LIHC(Liver hepatocellular carcinoma), LUAD(Lung adenocarcinoma), LUSC(Lung squamous cell carcinoma), PRAD(Prostate adenocarcinoma), STAD(Stomach adenocarcinoma), and UCEC(Pancreatic adenocarcinoma); significantly increased in CHOL(Cholangiocarcinoma), GBM(Glioblastoma multiforme), KIRP(Kidney renal papillary cell carcinoma), and THCA(Thyroid carcinoma); and had no significant effect on ESCA(2Esophageal carcinoma), HNSC(Head and Neck squamous cell carcinoma), LGG(Brain Lower Grade Glioma), and PAAD(Pancreatic adenocarcinoma) (**Figure 7A**). Meanwhile, SLCO5A1 was significantly lower in KICH(Kidney Chromophobe); significantly higher in BRCA(Breast invasive carcinoma), CHOL(Cholangiocarcinoma), COAD(Colon adenocarcinoma), ESCA(Esophageal carcinoma), KIRC(Kidney renal clear cell carcinoma), KIRP(Kidney renal papillary cell carcinoma), LGG(Brain Lower Grade Glioma), LIHC(Liver hepatocellular carcinoma), LUAD(Lung adenocarcinoma), LUSC(Lung squamous cell carcinoma), READ(Rectum adenocarcinoma), STAD(Stomach adenocarcinoma), and UCEC(Uterine Corpus Endometrial Carcinoma); and significantly higher in BLCA(Bladder Urothelial Carcinoma), GBM(Glioblastoma multiforme), HNSC(Head and Neck squamous cell carcinoma), PAAD(Pancreatic adenocarcinoma), PRAD(Prostate adenocarcinoma), and THCA(Thyroid carcinoma) (**Figure 7B**). Finally, AZGP1 was significantly decreased in BLCA(Bladder Urothelial Carcinoma), CHOL(Cholangiocarcinoma), ESCA(Esophageal carcinoma), HNSC(Head and Neck squamous cell carcinoma), KIRC(Kidney renal clear cell carcinoma), KIRP(Kidney renal papillary cell carcinoma), LIHC(Liver hepatocellular carcinoma), LUAD(Lung adenocarcinoma), LUSC(Lung squamous cell carcinoma), THCA, and UCEC(Pancreatic adenocarcinoma); significantly increased in COAD(Colon adenocarcinoma) and READ(Rectum adenocarcinoma); and had no significant effect on BRCA(Breast invasive carcinoma), GBM (Glioblastoma multiforme), KICH(Kidney Chromophobe), LGG(Brain Lower Grade Glioma), PAAD(Pancreatic adenocarcinoma), and STAD(Stomach adenocarcinoma) **Figure 7C**. Subsequently, we evaluated the prognostic effects of the three core genes on these cancers and found that CTF1 had a significant effect Htabon the prognosis of ACC(Adrenocortical carcinoma), COAD(Colon adenocarcinoma), ESCA(Esophageal carcinoma), GBM(Glioblastoma multiforme), LGG(Brain Lower Grade Glioma), LUAD(Lung adenocarcinoma), MESO(Mesothelioma), UCEC(Pancreatic adenocarcinoma), and UVM(Uveal Melanoma); SLCO5A1 had a significant effect on the prognosis of KIRC(Kidney renal clear cell carcinoma), LGG, PAAD(Pancreatic adenocarcinoma), and UVM(Uveal Melanoma); and AZGP1 had a significant effect on the prognosis of KIRC(Kidney renal clear cell carcinoma), KIRP(Kidney renal papillary cell carcinoma), UCEC(Pancreatic adenocarcinoma), and UVM(Uveal Melanoma) (**Figures 7D–F**, all *p* < 0.05).

## Discussion

In this study, we obtained two subgroups of UM through hierarchical clustering for which we performed differential gene analysis, as well as GO, KEGG, and Hallmark enrichment analyses focused on the immune response signaling pathways. Subsequent prognostic survival analysis of these differential genes identified 53 immune prognostic factors. Three core genes, namely, CTF1, SLCO5A1, and AZGP1, were obtained for the LASSO construct risk model. The pathway correlation analysis demonstrated that these three factors were associated with the immune response. Pan-cancer analysis further demonstrated the heterogeneous role of these three core genes.

Identification of novel features in UM based on immune-related genes provides new directions for assessing immune efficacy. Further evaluation of these classifications based on genetic features may help to develop immunotherapeutic strategies and improve the sensitivity to different subtypes of UM. In this study, we analyzed TCGA dataset and classified patients into two different immune subtypes, with significant differences in prognostic and clinical characteristics between the two groups. Due to the heterogeneity in gene expression and prognosis between the two immune subtypes, we hypothesized that integrated tumor ICI analysis and immune gene expression pattern assessment would be a new approach to develop patient-tailored and customized treatment strategies.

Understanding the TME structures and applying the insights gained to drug design have become a hot topic in modern cancer research (13). CD8+ T cells have been found to have a favorable prognostic role in many cancers (14). The immunosuppressive factors present in the TME can suppress T cell-mediated responses and rewire their activity to benefit tumors in which T-cell infiltration is related to poor prognosis (15). Studies have shown that metalloproteinases are effective targets for cancer therapy (16). Aside from acting as simple effectors of angiogenesis and metastasis, they also play a role in the regulation of immune responses. It has recently been shown that UM high-risk populations are related to a significantly enhanced metalloproteinase profile, which may play a dominant role in driving UM metastasis. CTF1 is a mitogenic cytokine of the interleukin 6 family. Meanwhile, CTF1 contains the transmembrane and cytoplasmic structural domains of E-calmodulin and is produced upon cleavage. It can act as a cleavage product of E-calmodulin, in addition to CTF itself, which can also act as a downstream signaling molecule. Studies have shown that the MMP28–E-calmodulin–CTF association mechanism promotes colorectal cancer progression and metastasis (17).

One of the factors associated with increased immune infiltration is chromosome 3 monosomy, which is considered a negative prognostic factor, while the early acquisition of chromosome 8 is associated with macrophage infiltration (18). SLCO5A1 is located on chromosome 8, but it remains unknown whether it can influence chromosomal variants leading to the development and progression of UM. A recent study has shown that SLCO5A1 is a negative prognostic factor in UM. Moreover, in another study, AZGP1 was found to be a good prognostic factor, which coincides with our results, while SLCO5A1 and AZGP1 are closely associated with the abundance of neutrophils and CD8+ T cells (19).

The present study has some limitations. The current results need to be validated in immunotherapy clinical trials with larger UM cohorts, which will confirm the utility of classification in clinical evaluation and decision-making. In addition, the present study was based on transcriptome expression profiles of UM tissues from TCGA database and did not have a controlled study of normal uveal melanocytes, which may not allow for an accurate prediction. Therefore, it is important to better understand the circulating biomarkers released into the bloodstream from tumor cells and tumor-associated immune cells. Further *in vivo* and *in vitro* experiments should investigate the potential functional and mechanistic differences between subtypes. Finally, although we did a pan-cancer analysis of 3 core genes, the results of this study and ICI scores may be applicable to other cancers based on the heterogeneous functional role of genes in different cancers, which need further investigation.

In our study, we analyzed both the expression and prognosis of these three core genes in other cancers and found that CTF1, SLCO5A1, and AZGP1 presented different functional roles in other cancers. A recent study reported that the systematic screening of differentially expressed circRNAs (DEcircRNAs), miRNAs (DEmiRNAs), and mRNAs (DEGs) associated with LUAD identified a ceRNA prognostic regulatory network consisting of 1 circRNA, 2 miRNAs, and 7 mRNAs, in which CTF1 as a good prognostic gene is regarded as a drug target (20). In addition, it was shown that CTF1/N-Cad (CTF1) is a product of extracellular metalloproteinase (MMP), which cleaves near the interface between the extracellular and transmembrane regions of N-calcine mucin, and that the extracellular and intracellular cleavages of N-calcine mucin may be involved in elevated MMP-9 expression and enhanced invasion of human nasopharyngeal carcinoma cells (21). In some studies, the SNP(single nucleotide polymorphism) in SLCO5A1 was correlated with the clinical staging of prostate cancer (22). Similarly, in some studies, SLCO5A1 expression was found to decrease during differentiation from monocytes to macrophages but increase during differentiation from monocytes to mature dendritic cells (23). This differential expression profile may be related to the fact that SLCO5A1 plays an important role in the immune function involved in chylomicronoma. In our data, AZGP1 was considered to have a significant impact on KIRC, KIRP, UCEC, and UVM prognosis, but it has been shown that AZGP1 could be a feasible candidate biomarker for colorectal cancer based on an analysis of the GSE21962, GSE24551, and GSE29638 datasets in the GEO(Gene Expression Omnibus) database (24). Patients with oral squamous cell carcinoma (OSCC) have low salivary levels of AZGP1 (25)and low mRNA levels of AZGP1 in OSCC tumor tissues (26). The role of AZGP1 in inhibiting cell invasion and migration (27, 28) suggests its correlation with poor disease response. Similarly, the low mRNA/protein expression of AZGP1 is associated with disease progression and poor survival in pancreatic cancer (29, 30). The above data underscore the heterogeneous role that the three core genes, CTF1, SLCO5A1, and AZGP1, present in other cancers.

In summary, in our study, based on TCGA database, we obtained three core immune-related prognostic factors for UM, namely, CTF1, SLCO5A1, and AZGP1, through bioinformatics methods. Notably, the immune infiltration analysis proved that the risk model that we constructed was related to B cells, myeloid dendritic cells, and CD8+ T cells and that the three core genes mentioned above were mainly the focus of immune-related pathway signaling. These results could drive new therapies for UM treatment.

## Data availability statement

The original contributions presented in the study are included in the article/**Supplementary Material**. Further inquiries can be directed to the corresponding author.

## Author contributions

All authors listed have made a substantial, direct, and intellectual contribution to the work, and approved it for publication.

## Funding

The project was sponsored by grants from Hunan Provincial Natural Science Foundation of China (2020JJ4912).

## Conflict of interest

The authors declare that the research was conducted in the absence of any commercial or financial relationships that could be construed as a potential conflict of interest.

## Publisher’s note

All claims expressed in this article are solely those of the authors and do not necessarily represent those of their affiliated organizations, or those of the publisher, the editors and the reviewers. Any product that may be evaluated in this article, or claim that may be made by its manufacturer, is not guaranteed or endorsed by the publisher.

## Abbreviations

BLCA, Bladder Urothelial Carcinoma; BRCA, Breast invasive carcinoma; CHOL, Cholangiocarcinoma; COAD, Colon adenocarcinoma; ESCA, Esophageal carcinoma; GBM, Glioblastoma multiforme; HNSC, Head and Neck squamous cell carcinoma; KICH, Kidney Chromophobe KIRC Kidney renal clear cell carcinoma; LGG, Brain Lower Grade Glioma; LIHC, Liver hepatocellular carcinoma; LUAD, Lung adenocarcinoma; LUSC, Lung squamous cell carcinoma; PAAD, Pancreatic adenocarcinoma; PRAD, Prostate adenocarcinoma; READ, Rectum adenocarcinoma; STAD, Stomach adenocarcinoma; THCA, Thyroid carcinoma; UCEC, Uterine Corpus Endometrial Carcinoma.

## References

[1] Amaro A Gangemi R Piaggio F Angelini G Barisione G Ferrini S. The biology of uveal melanoma. Cancer Metastasis Rev (2017) 36(1):109–40. doi: 10.1007/s10555-017-9663-3 PMC5385203 28229253

[2] Kaliki S Shields CL. Uveal melanoma: Relatively rare but deadly cancer. Eye (Lond) (2017) 31(2):241–57. doi: 10.1038/eye.2016.275 PMC5306463 27911450

[3] Stalhammar G. Forty-year prognosis after plaque brachytherapy of uveal melanoma. Sci Rep (2020) 10(1):11297. doi: 10.1038/s41598-020-68232-7 32647177 PMC7347921

[4] Rantala ES Hernberg M Kivela TT. Overall survival after treatment for metastatic uveal melanoma: A systematic review and meta-analysis. Melanoma Res (2019) 29(6):561–8. doi: 10.1097/CMR.0000000000000575 PMC6887637 30664106

[5] Rodriguez-Vidal C Fernandez-Diaz D Fernandez-Marta B Lago-Baameiro N Pardo M Silva P. Treatment of metastatic uveal melanoma: Systematic review. Cancers (Basel) (2020) 12(9):2557. doi: 10.3390/cancers12092557 PMC7565536 32911759

[6] Jager MJ Dogrusoz M Woodman SE. Uveal melanoma: Identifying immunological and chemotherapeutic targets to treat metastases. Asia Pac J Ophthalmol (Phila) (2017) 6(2):179–85. doi: 10.22608/APO.201782 28399339

[7] Ravindran S Rasool S Maccalli C. The cross talk between cancer stem Cells/Cancer initiating cells and tumor microenvironment: The missing piece of the puzzle for the efficient targeting of these cells with immunotherapy. Cancer Microenviron (2019) 12(2-3):133–48. doi: 10.1007/s12307-019-00233-1 PMC6937350 31758404

[8] Cully M. Tumour microenvironment: Fibroblast subtype provides niche for cancer stem cells. Nat Rev Cancer (2018) 18(3):136. doi: 10.1038/nrc.2018.18 29467526

[9] Gezgin G Luk SJ Cao J Dogrusöz M van der Steen DM Hagedoorn RS. PRAME as a potential target for immunotherapy in metastatic uveal melanoma. JAMA Ophthalmol (2017) 135(6):541–9. doi: 10.1001/jamaophthalmol.2017.0729 PMC5509351 28448663

[10] Wilkerson MD Hayes DN. ConsensusClusterPlus: A class discovery tool with confidence assessments and item tracking. Bioinformatics (2010) 26(12):1572–3. doi: 10.1093/bioinformatics/btq170 PMC2881355 20427518

[11] Tao C Huang K Shi J Hu Q Li K Zhu X.. Genomics and prognosis analysis of epithelial-mesenchymal transition in glioma. Front Oncol (2020) 10:183. doi: 10.3389/fonc.2020.00183 32154177 PMC7047417

[12] Yu P Tong L Song Y Qu H Chen Y. Systematic profiling of invasion-related gene signature predicts prognostic features of lung adenocarcinoma. J Cell Mol Med (2021) 25(13):6388–402. doi: 10.1111/jcmm.16619 PMC8256358 34060213

[13] Smit KN Jager MJ de Klein A Kiliҫ E. Uveal melanoma: Towards a molecular understanding. Prog Retin Eye Res (2020) 75:100800. doi: 10.1016/j.preteyeres.2019.100800 31563544

[14] Talmadge JE. Immune cell infiltration of primary and metastatic lesions: Mechanisms and clinical impact. Semin Cancer Biol (2011) 21(2):131–8. doi: 10.1016/j.semcancer.2010.12.002 21145968

[15] Kaliki S Shields CL Shields JA. Uveal melanoma: Estimating prognosis. Indian J Ophthalmol (2015) 63(2):93–102. doi: 10.4103/0301-4738.154367 25827538 PMC4399142

[16] Webb AH Gao BT Goldsmith ZK Irvine AS Saleh N Lee RP. Inhibition of MMP-2 and MMP-9 decreases cellular migration, and angiogenesis in in vitro models of retinoblastoma. BMC Cancer (2017) 17(1):434. doi: 10.1186/s12885-017-3418-y 28633655 PMC5477686

[17] Drury J Rychahou PG Kelson CO Geisen ME Wu Y He D. Upregulation of CD36, a fatty acid translocase, promotes colorectal cancer metastasis by increasing MMP28 and decreasing e-cadherin expression. Cancers (Basel) (2022) 14(1):252. doi: 10.3390/cancers14010252 35008415 PMC8750155

[18] Gezgin G Dogrusöz M van Essen TH Kroes WGM Luyten GPM van der Velden PA. Genetic evolution of uveal melanoma guides the development of an inflammatory microenvironment. Cancer Immunol Immunother (2017) 66(7):903–12. doi: 10.1007/s00262-017-1991-1 PMC5489616 28391358

[19] Luo H Ma C. Identification of prognostic genes in uveal melanoma microenvironment. PloS One (2020) 15(11):e0242263. doi: 10.1371/journal.pone.0242263 33196683 PMC7668584

[20] Gao L Zhang L. Construction and comprehensive analysis of a ceRNA network to reveal potential prognostic biomarkers for lung adenocarcinoma. BMC Cancer (2021) 21(1):849. doi: 10.1186/s12885-021-08462-8 34301211 PMC8299662

[21] Hsu CC Huang SF Wang JS Chu WK Nien JE Chen WS. Interplay of n-cadherin and matrix metalloproteinase 9 enhances human nasopharyngeal carcinoma cell invasion. BMC Cancer (2016) 16(1):800. doi: 10.1186/s12885-016-2846-4 27737648 PMC5064931

[22] Perlman D Chikarmane H Halvorson HO. Improved resolution of DNA fragments in polysaccharide-supplemented agarose gels. Anal Biochem (1987) 163(1):247–54. doi: 10.1016/0003-2697(87)90120-5 3619025

[23] Sebastian K Detro-Dassen S Rinis N Fahrenkamp D Müller-Newen G Merk HF. Characterization of SLCO5A1/OATP5A1, a solute carrier transport protein with non-classical function. PloS One (2013) 8(12):e83257. doi: 10.1371/journal.pone.0083257 24376674 PMC3869781

[24] Ding D Han S Zhang H He Y Li Y. Predictive biomarkers of colorectal cancer. Comput Biol Chem (2019) 83:107106. doi: 10.1016/j.compbiolchem.2019.107106 31542707

[25] Jain A Kotimoole CN Ghoshal S Bakshi J Chatterjee A Prasad TSK. Identification of potential salivary biomarker panels for oral squamous cell carcinoma. Sci Rep (2021) 11(1):3365. doi: 10.1038/s41598-021-82635-0 33564003 PMC7873065

[26] Suhr ML Dysvik B Bruland O Warnakulasuriya S Amaratunga AN Jonassen I. Gene expression profile of oral squamous cell carcinomas from Sri Lankan betel quid users. Oncol Rep (2007) 18(5):1061–75. 17914555

[27] Feng M Feng J Chen W Wang W Wu X Zhang J. Lipocalin2 suppresses metastasis of colorectal cancer by attenuating NF-kappaB-Dependent activation of snail and epithelial mesenchymal transition. Mol Cancer (2016) 15(1):77. doi: 10.1186/s12943-016-0564-9 27912767 PMC5135816

[28] Kong B Michalski CW Hong X Valkovskaya N Rieder S Abiatari I. AZGP1 is a tumor suppressor in pancreatic cancer inducing mesenchymal-to-Epithelial transdifferentiation by inhibiting TGF-Beta-Mediated ERK signaling. Oncogene (2010) 29(37):5146–58. doi: 10.1038/onc.2010.258 20581862

[29] Burdelski C Kleinhans S Kluth M Hube-Magg C Minner S Koop C. Reduced AZGP1 expression is an independent predictor of early PSA recurrence and associated with ERG-fusion positive and PTEN deleted prostate cancers. Int J Cancer (2016) 138(5):1199–206. doi: 10.1002/ijc.29860 26383228

[30] Zhang AY Grogan JS Mahon KL Rasiah K Sved P Eisinger DR. A prospective multicentre phase III validation study of AZGP1 as a biomarker in localized prostate cancer. Ann Oncol (2017) 28(8):1903–9. doi: 10.1093/annonc/mdx247 28486686

