# Supplementary material for: The prognostic value of immune-related genes AZGP1, SLCO5A1, and CTF1 in Uveal melanoma
Source: Front Oncol. 2022 Aug 16;12:918230. doi: 10.3389/fonc.2022.918230 (PMC9425775; doi:10.3389/fonc.2022.918230)
Supplement: Supplementary file 1 [file DataSheet_1.zip › Table 1.docx]

Table s1 Pathway correlation analysis was performed on CTF1

| Pathway | *p* | Spearman | CI95% |
| --- | --- | --- | --- |
| Tumor Inflammation Signature | 8.04e−06 | −0.48 | −0.63, −0.28 |
| Cellular response to hypoxia | 7.51e−07 | −0.52 | −0.67, −0.33 |
| Tumor proliferation signature | 2.09e−04 | −0.40 | −0.58, −0.19 |
| EMT markers | 1e−05 | −0.47 | −0.63, −0.28 |
| ECM-relatted genes | 0.688 | −0.05 | −0.27, 0.18 |
| Angiogenesis | 7.35e−06 | −0.48 | −0.64, −0.28 |
| Apoptosis | 4.19e−07 | −0.53 | −0.68, −0.35 |
| DNA repair | 0.235 | −0.13 | −0.35, 0.09 |
| G2M checkpoint | 2.37e−06 | −0.50 | −0.65, −0.31 |
| Inflammatory response | 3.44e−06 | −0.49 | −0.65, −0.30 |
| PI3K AKT mTOR pathway | 7.28e−11 | −0.65 | −0.76, −0.50 |
| P53 pathway | 0.001 | −0.35 | −0.53, −0.14 |
| MYC targets | 0.001 | −0.37 | −0.55, −0.16 |
| TGFB | 2.24e−06 | −0.50 | −0.65, −0.31 |
| IL-10 Anti-inflammatory Signaling Pathway | 5.26e−07 | −0.53 | −0.67, −0.34 |
| Genes up-regulated by reactive oxigen species (ROS) | 4.11e−11 | −0.66 | −0.77, −0.50 |
| DNA replication | 2.04e−04 | −0.40 | −0.58, −0.20 |
| Collagen formation | 4.2e−04 | −0.39 | −0.56, −0.17 |
| Degradation of ECM | 1.22e−04 | −0.42 | −0.59, −0.21 |
