# Supplementary material for: The prognostic value of immune-related genes AZGP1, SLCO5A1, and CTF1 in Uveal melanoma
Source: Front Oncol. 2022 Aug 16;12:918230. doi: 10.3389/fonc.2022.918230 (PMC9425775; doi:10.3389/fonc.2022.918230)
Supplement: Supplementary file 1 [file DataSheet_1.zip › Table 2.docx]

Table s2 Pathway correlation analysis was performed on SLCO5A1

| Pathway | *p* | Spearman | CI95% |
| --- | --- | --- | --- |
| Tumor Inflammation Si | 0.003 | 0.32 | 0.10, 0.51 |
| Cellular response to hypoxia | 1.08e−05 | 0.47 | 0.27, 0.63 |
| Tumor proliferation signature | 0.009 | 0.29 | 0.07, 0.49 |
| EMT markers | 3.37e−04 | 0.39 | 0.18, 0.57 |
| ECM−relatted genes | 0.572 | −0.06 | −0.29, 0.16 |
| Angiogenesis | 2.6e−05 | 0.45 | 0.25, 0.61 |
| Apoptosis | 5.85e−06 | 0.48 | 0.29, 0.64 |
| DNA repair | 0.207 | −0.14 | −0.36, 0.09 |
| G2M checkpoint | 4.35e−05 | 0.44 | 0.24, 0.61 |
| Inflammatory response | 2.16e−04 | 0.4 | 0.19, 0.58 |
| PI3K AKT mTOR pathway | 8e−09 | 0.59 | 0.42, 0.72 |
| P53 pathway | 0.033 | 0.24 | 0.01, 0.44 |
| MYC targets | 2.04e−04 | 0.4 | 0.20, 0.58 |
| TGFB | 2.73e−05 | 0.45 | 0.25, 0.61 |
| IL−10 Anti−inflammatory Signaling Pathway | 2.85e−04 | 0.4 | 0.19, 0.57 |
| Genes up−regulated by reactive oxigen species (ROS) | 3.32e−07 | 0.53 | 0.35, 0.68 |
| DNA replication | 0.008 | 0.29 | 0.07, 0.49 |
| Collagen formation | 0.016 | 0.27 | 0.05, 0.47 |
