# Supplementary material for: The prognostic value of immune-related genes AZGP1, SLCO5A1, and CTF1 in Uveal melanoma
Source: Front Oncol. 2022 Aug 16;12:918230. doi: 10.3389/fonc.2022.918230 (PMC9425775; doi:10.3389/fonc.2022.918230)
Supplement: Supplementary file 1 [file DataSheet_1.zip › Table 3.docx]

Table s3 Pathway correlation analysis was performed on AZGP1

| Pathway | *p* | Spearman | CI95% |
| --- | --- | --- | --- |
| Tumor Inflammation Signature | 3.46e−09 | −0.60 | −0.73, −0.44 |
| Cellular response to hypoxia | 7.4e−09 | −0.59 | −0.72, −0.42 |
| Tumor proliferation signature | 0.001 | −0.38 | −0.55, −0.16 |
| EMT markers | 3.19e−06 | −0.49 | −0.65, −0.30 |
| ECM-relatted genes | 0.144 | −0.16 | −0.38, 0.06 |
| Angiogenesis | 1.64e−05 | −0.46 | −0.62, −0.26 |
| Apoptosis | 4.23e−11 | −0.66 | −0.77, −0.50 |
| DNA repair | 0.6 | −0.06 | −0.28, 0.17 |
| G2M checkpoint | 1.07e−04 | −0.42 | −0.59, −0.21 |
| Inflammatory response | 5.07e−10 | −0.63 | −0.75, −0.47 |
| PI3K AKT mTOR pathway | 1.84e−12 | −0.69 | −0.79, −0.55 |
| P53 pathway | 1.9e−05 | −0.46 | −0.62, −0.26 |
| MYC targets | 0.026 | −0.25 | −0.45, −0.02 |
| TGFB | 6.43e−06 | −0.48 | −0.64, −0.29 |
| IL-10 Anti-inflammatory Signaling Pathway | 1.59e−10 | −0.64 | −0.76, −0.48 |
| Genes up-regulated by reactive oxigen species (ROS) | 3.77e−12 | −0.68 | −0.79, −0..54 |
| DNA replication | 0.005 | −0.31 | −0.50, −0.09 |
| Collagen formation | 0.001 | −0.37 | −0.55, −0.15 |
